# Jarisch-Herxheimer reaction as a complication of penicillin G and ceftriaxone treatment in neurosyphilis

**DOI:** 10.1186/s12879-026-12683-2

**Published:** 2026-01-27

**Authors:** Xin Feng, Junjun Yu, Xin Gu, Yilan Yang, Nanyan Jiang, Guixuan Wang, Danyang Zou, Meiping Ye, Pingyu Zhou

**Affiliations:** 1 STD Institute, Shanghai Skin Disease Hospital, School of Medicine, Tongji University, Shanghai, 200443 China; 2 Department of Dermatology, Xinhua Hospital, Shanghai Jiaotong University School of Medicine, Shanghai, 200092 China

**Keywords:** Ceftriaxone, Clinical pattern, Jarisch-Herxheimer reaction, Neurosyphilis, Penicillin G

## Abstract

**Background:**

Jarisch-Herxheimer reaction (JHR) is a complication of antibiotic treatment in neurosyphilis, and the profile of JHR in patients treated with different antibiotics remains unclear. This study investigates the incidence, clinical manifestations, and management of JHR in patients treated with penicillin G or ceftriaxone.

**Methods:**

A retrospective study was conducted at the Shanghai Skin Disease Hospital, China, involving 1010 inpatients diagnosed with neurosyphilis, among whom 103 developed JHR. Statistical analyses included Student’s t-test or Mann-Whitney test for continuous variables and Fisher’s exact test for categorical data.

**Results:**

The overall incidence of JHR among neurosyphilis patients was 10.20% (103/1010), with rates varying from 0% − 25% across different subtypes. Incidence was similar in penicillin G-treated (10.20%, 92/902) and ceftriaxone-treated (10.19%, 11/108) cohorts. The 92 JHR cases of penicillin G group manifested as fever (98.91%, 91/92) and neuropsychiatric symptoms (25%, 23/92). All 11 cases of JHR in the ceftriaxone group presented with fever (100%, 11/11). Penicillin G treatment resulted in an earlier fever onset (8.92 ± 4.98 h vs. 14.00 ± 5.90 h, 95% CI: 1.861 to 8.303, *P* < 0.05) and a shorter time to defervescence (16.35 ± 5.48 h vs. 20.80 ± 3.79 h, 95% CI: 1.085 to 7.848, *P* < 0.05) than ceftriaxone. Management strategies included physical cooling, indomethacin suppositories, and supportive treatment for neuropsychiatric symptoms. Neuropsychiatric symptoms of JHR resolved within 2–7 days of initiating antibiotic therapy.

**Conclusions:**

JHR is a notable complication of neurosyphilis treatment, presenting as febrile and neuropsychiatric symptoms. JHR frequency is comparable between penicillin G and ceftriaxone treatment. Penicillin G triggers an earlier febrile response of JHR compared to ceftriaxone. While generally mild and self-limiting, JHR can present with neuropsychiatric symptoms and severe conditions. This study contributes to the understanding of JHR and provides insights into the comparative effects of penicillin G and ceftriaxone treatment in neurosyphilis.

**Clinical trial:**

Not applicable.

## Background

Syphilis, a curable sexually transmitted disease caused by the bacterium *Treponema pallidum*, can lead to multisystem involvement. Neurosyphilis results from the invasion of the central nervous system (CNS) by *Treponema pallidum* and it can occur at any stage of syphilis. After reaching historically low rates in the 2000s, syphilis has re-emerged as a major global health concern [1]. In 2022, World Health Organization (WHO) estimated 8 million new cases among adults aged 15–49 worldwide, with annual increases since 2016 [2]. European Centre for Disease Prevention and Control (ECDC) reported a crude syphilis notification rate of 8.5 cases per 100,000 population in 2022, marking a 41% rise from 2018 across 29 European Union/European Economic Area (EU/EEA) Member States [3]. Similarly, in the United States, syphilis cases reached 209,253 in 2023-the highest number reported since 1950, reflecting a 57.2% increase from 2019 to 2023 [4]. Neurosyphilis remains a persistent public health challenge, with an estimated global incidence of 0.16–2.47 cases per 100,000 population [5, 6].

Current guidelines from Europe, the United States, Germany and China recommend benzylpenicillin (penicillin G) as the first-line therapy for neurosyphilis (18–24 million units IV daily, administered as 3–4 million units every 4 h for 10–14 days), while ceftriaxone (1–2 g IV daily for 10–14 days) is suggested as an alternative [7–10]. A notable complication of neurosyphilis treatment is the Jarisch-Herxheimer reaction (JHR), a transient and self-limiting inflammatory response. Recent studies showed a JHR incidence of 9.3% in neurosyphilis, with historical variability ranging from 5.2% to 54% in the 1950s [11–13]. JHR typically manifests within 4 to 5 h of antibiotic administration and resolves within 24 h [11, 13]. Clinical symptoms include fever, headache, myalgia, chills and rigours, as well as neuropsychiatric symptoms such as confusion, agitation, hallucinations, convulsions, memory impairment [11, 14]. Although rare, severe cases of JHR can be life-threatening, warranting careful monitoring during treatment [15–18].

JHR is an important complication of antibiotic treatment in neurosyphilis, but its clinical profile in patients treated with different antibiotics remains unclear. While penicillin is the first-line treatment, previous studies suggested it may cause a higher frequency of JHR compared to other antibiotics in early syphilis, raising concerns about its impact in neurosyphilis [19–21]. Furthermore, ceftriaxone has been reported to be as effective as penicillin G in the treatment of neurosyphilis [22]. Thus, there is great significance to systematically compare the frequency and severity of JHR between the two antibiotic regimens in neurosyphilis. This study aims to detail the incidence, clinical manifestations, management of JHR in neurosyphilis patients treated with penicillin G versus ceftriaxone.

## Methods

### Setting and study participants

This retrospective study was conducted at the Shanghai Skin Disease Hospital, one of the largest neurosyphilis treatment centers worldwide. From 1 June 2017 to 28 February 2022, a total of 1010 inpatients meeting the following inclusion criteria were enrolled: (1) aged over 18 years; (2) confirmed diagnosis of neurosyphilis; (3) first episode of neurosyphilis treatment. Among these patients, 103 experienced a JHR.

Neurosyphilis was defined based on established sexually transmitted disease (STD) guidelines [7–9]: (1) Confirmed diagnosis of syphilis: both positive results of Treponema Pallidum Particle Agglutination (TPPA) test and Toluidine Red Unheated Serum Test (TRUST) in peripheral blood; (2) Reactive cerebrospinal fluid (CSF)-Venereal Disease Research Laboratory (VDRL) and CSF-TPPA tests; (3)in case with nonreactive CSF-VDRL but positive CSF-TPPA, a CSF protein concentration > 500 mg/L and/or white blood cell (WBC) count ≥ 10 cells/µl, with no other identifiable causes.

Patients were classified according to clinical manifestations of neurosyphilis [7], based on established clinical syndromes such as: (1) Asymptomatic neurosyphilis: no clinical neuropsychiatric symptoms; (2) Meningitis: fever, headache, nausea, vomiting, papilledema, neck stiffness, positive meningeal irritation signs, and other meningitis symptoms; (3) Meningovascular neurosyphilis: occlusive cerebrovascular syndrome including hemiplegia, aphasia, epileptic seizures when the brain is involved, and spinal cord infarction when the spinal cord is involved; (4) Gummatous syphilis: (i) Cerebral gumma: intracranial tumor-like symptoms, including signs of increased intracranial pressure (headache, nausea, vomiting, papilledema and neck stiffness) and epileptic seizures; (ii) Spinal cord gumma: paraplegia, urinary and fecal incontinence, and sensory loss below the level of the lesion; (5) General paresis: psychiatric and behavioral disturbances such as increasing cognitive deficits, weakness of discrimination and judgment, psychotic episodes, speech disorders, abnormal pupillary reaction, epileptic seizures, reflex anomalies, dementia, and marasmus; (6) Tabes dorsalis: The lesions involve the dorsal columns and posterior nerve roots of the spinal cord. Symptoms include sensory ataxia and stabbing pain, Argyll Robertson pupils, lightning-like pains in the lower limbs, paresthesia or hypoesthesia, decreased or absent tendon reflexes, decreased muscle tone in the lower limbs, urinary retention, Charcot arthropathy.

The standard treatment regimen was aqueous crystalline penicillin G (4 million units every 4 h for 14 days, IV), and ceftriaxone sodium (1 g every 12 h for 14 days, IV) was used as an alternative in penicillin G-allergic patients. JHR was defined as the occurrence of one or more of the following symptoms after antibiotic administration: fever (t ≥ 37.2 °C), chill, headache, convulsions, mania, consciousness disorder, or other exacerbated or new neuropsychiatric symptoms.

### Data collection

Data were extracted from Shanghai Skin Disease Hospital Information System and Nursing Information System. Demographic data included gender, age, clinical manifestations and diagnosis. Laboratory tests included plasma TRUST titre, CSF VDRL titres, white blood cell (WBC) count and protein concentration. Clinical information included antibiotic therapy details, JHR manifestations, JHR management strategies and treatment outcomes.

### Statistical analysis

All statistical analyses and visualizations were performed using GraphPad Prism (version 10.6). Normality of continuous variables was assessed using the Shapiro–Wilk test. Continuous variables following a normal distribution were presented as mean ± standard deviation (SD) and were compared using Student’s t-test; non-normally distributed continuous variables were presented as median with interquartile range [IQR] and were compared using the Mann–Whitney U test. Categorical variables are presented as counts and percentages, and were compared using Fisher’s exact test. For time-to-event variables (e.g., time to fever onset, time to defervescence), analyses were restricted to patients who experienced the event (e.g., analysis of fever timing was performed among patients who developed fever). Where appropriate, effect sizes with 95% confidence intervals (CIs) are reported. A two-sided *P* value < 0.05 was considered statistically significant.

### Ethical considerations

This study was conducted in accordance with the Declaration of Helsinki and was approved by the Ethics Committee of Shanghai Skin Disease Hospital (no. 2020-27). Given its retrospective nature, the Ethics Committee waived the requirement for informed consent. All patient data were anonymized before analysis.

## Results

### Occurrence rate of JHR in penicillin G and ceftriaxone treatment cohort

**Fig. 1:**
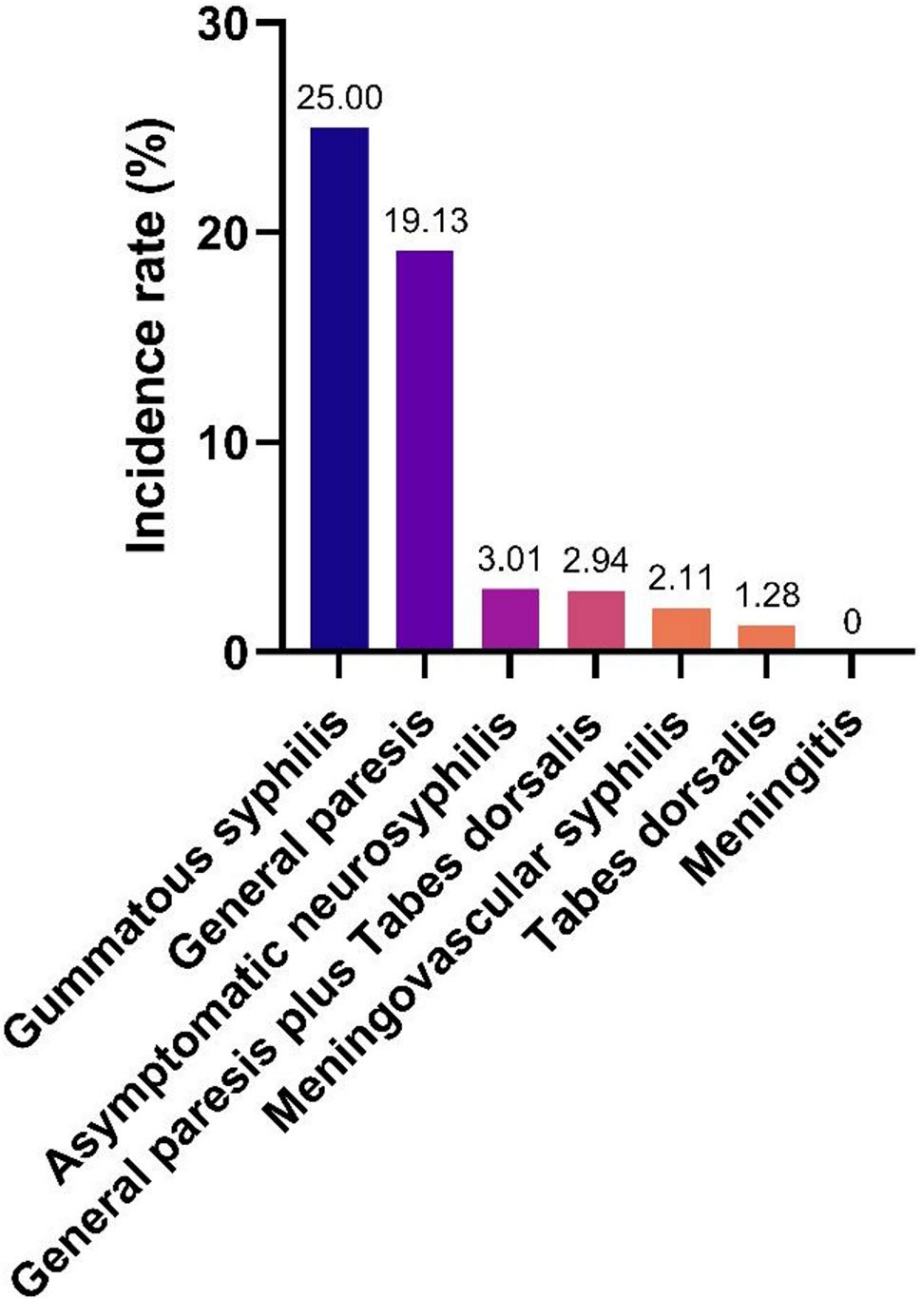
Occurrence rate of Jarisch-Herxheimer reaction in various neurosyphilis types

Between 1 June 2017 and 28 February 2022, 1010 patients were diagnosed with neurosyphilis at Shanghai Skin Disease Hospital. The overall incidence of Jarisch-Herxheimer reaction in neurosyphilis was estimated to be 10.20% (103/1,010). Incidence ranged from 0% to 25% among different neurosyphilis subtypes, 25.00% (1/4) in gummatous syphilis, followed by 19.13% (88/460) in general paresis, 3.01% (10/332) in asymptomatic neurosyphilis, 2.94% (1/34) in general paresis plus tabes dorsalis, 2.11% (2/95) in meningovascular syphilis, 1.28% (1/78) in tabes dorsalis, 0% (0/7) in meningitis (Fig. 1).

**Fig. 2:**
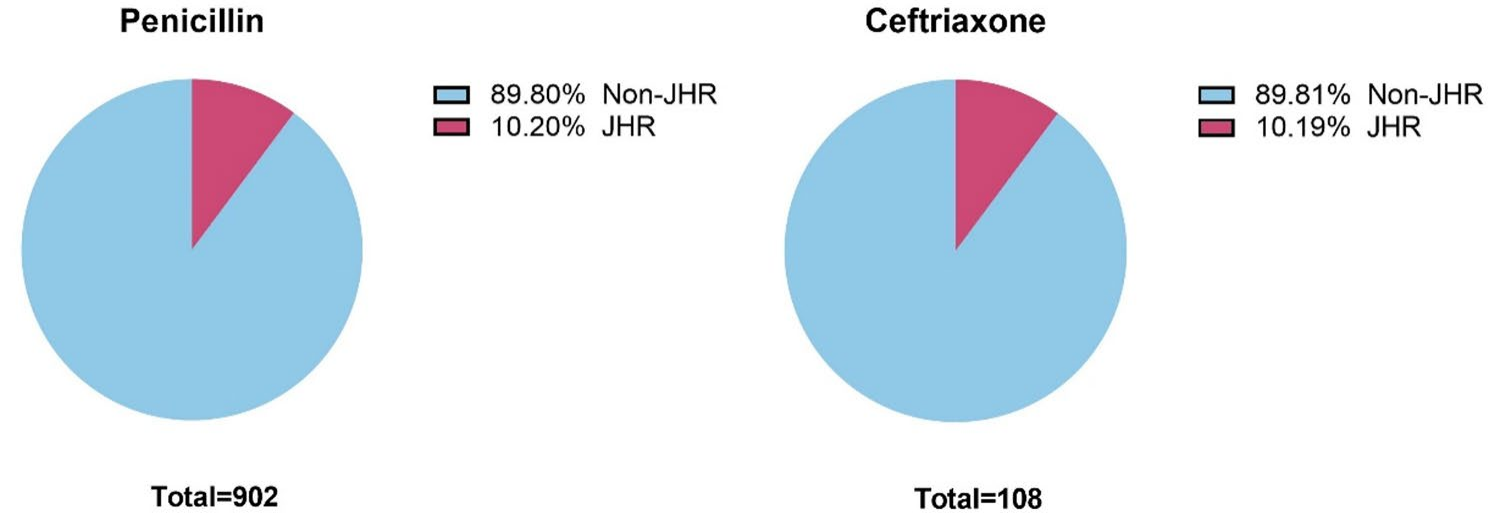
Occurrence rate of JHR in penicillin G and ceftriaxone treatment cohort

Of the enrolled 1010 patients, 902 received penicillin G treatment, while 108 penicillin G-allergic cases were treated with ceftriaxone. JHR incidence was nearly identical between the two groups: 10.2% (92/902) in penicillin G cohort and 10.19% (11/108) in ceftriaxone cohort, with no significant statistical difference (*P* = 0.99) (Fig. 2).

### Baseline characteristics of the 103 JHR patients

Baseline demographic data (Table 1) showed comparable characteristics between JHR patients treated with penicillin G (*n* = 92) and ceftriaxone (*n* = 11), including sex distribution (77.2% vs. 72.7% male) and median age (57 years in both groups). There was a HIV-positive patient in the penicillin G arm. General paresis was the most common neurosyphilis subtype (87.0% vs. 72.7%), followed by asymptomatic neurosyphilis (9.8% vs. 9.1%). The laboratory findings were similar in terms of peripheral blood TRUST titre (1:48 vs. 1:32), CSF VDRL (1:8, both) and WBC count (44 vs.48). CSF protein concentration was higher in the penicillin G group (1,187 vs. 794, *P* < 0.05).

**Table 1:** Baseline data of 103 JHR patients under penicillin G and ceftriaxone treatment

Variable	Antibiotic treatment		P value
	Penicillin G (*N* = 92)	Ceftriaxone (*N* = 11)	
Persons living with HIV, n (%)	1 (1.1%)	0	
Male, n (%)	71(77.2%)	8 (72.7%)	0.715
Age, median [IQR]	57 [52-62.75]	57 [46–69]	0.767
Types of neurosyphilis, n (%)			
General paresis	80 (87.0%)	8 (72.7%)	0.199
Asymptomatic neurosyphilis	9 (9.8%)	1 (9.1%)	> 0.999
Meningovascular syphilis	1 (1.1%)	1 (1.1%)	
Tabes dorsalis	1 (1.1%)	0	
General paresis plus Tabes dorsalis	0	1 (1.1%)	
Gummatous syphilis	1 (1.1%)	0	
Peripheral blood plasma			
TRUST titre, median [IQR]	1:48 [1:16 − 1:112]	1:32 [1:32 − 1:64]	0.851
Cerebrospinal fluid			
VDRL titer, median [IQR]	1:8 [1:4 − 1:16]	1:8 [1:2 − 1:8]	0.131
WBC count (cells/µl), median [IQR]	44.0 [20.5–70.0]	48.0 [22.0-100.0]	0.645
Protein concentration (mg/L), median [IQR]	1187 [916.5–1518]	794 [738–1154]	0.013

Normality test was conducted for all continuous variables. The titres of plasma TRUST, CSF VDRL were normalized by log2 transformation. Student’s t or Mann-Whitney test was performed for continuous data appropriately. Fisher’s exact test in categorical data. Abbreviations: SD, standard deviation; IQR, interquartile range; TRUST, toluidine red unheated serum test; VDRL, venereal disease research laboratory test; WBC, white blood cell; JHR, Jarisch-Herxheimer reaction

### Clinical manifestations of JHR in penicillin G and ceftriaxone cohort

**Fig. 3:**
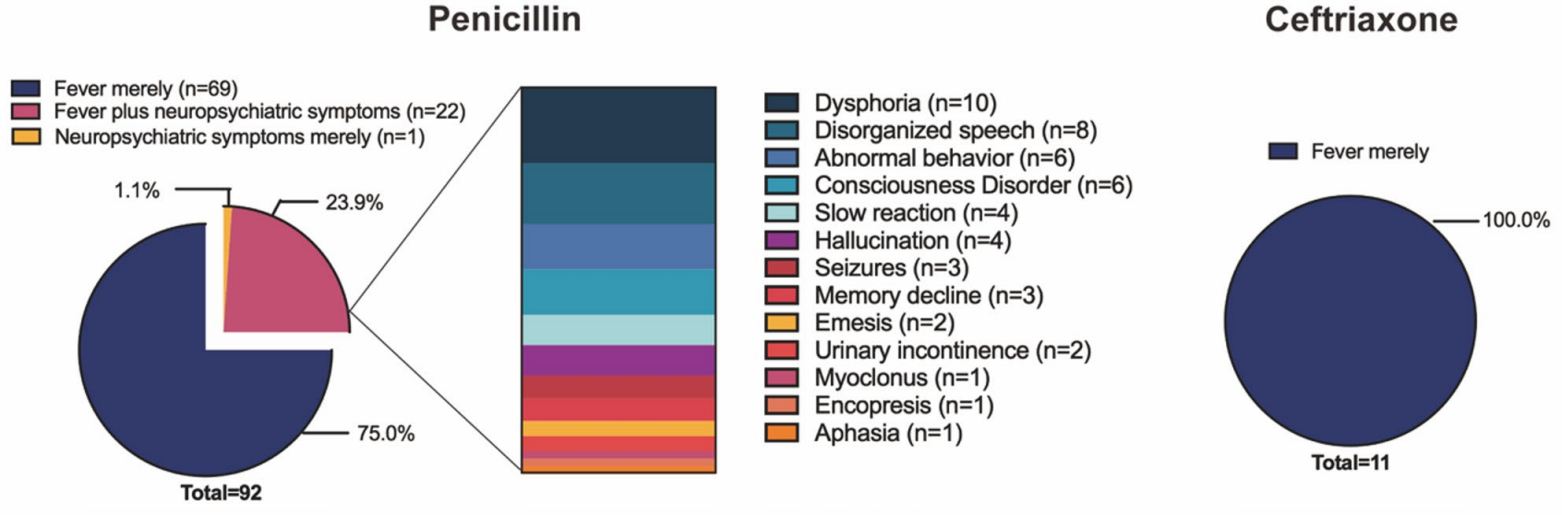
Symptoms of JHR in penicillin G and ceftriaxone treatment cohort

The 92 JHR cases of penicillin G group manifested as fever (98.9%, 91/92) and neuropsychiatric symptoms (25%, 23/92). All 11 cases of JHR in the ceftriaxone group presented with fever (100%, 11/11), with no neuropsychiatric symptoms observed (Fig. 3). The incidence rate of fever (98.9% vs. 100%) and neuropsychiatric symptoms (25% vs. 0%) in penicillin G group and ceftriaxone group did not differ significantly (*P* > 0.05). Notably, all the 23 neuropsychiatric JHR patients were associated with general paresis.

Fever developed within 9.47 ± 5.29 h, reached a peak temperature of 38.81 ± 0.62 °C around 10.41 ± 4.80 h, and resolved in 16.83 ± 5.49 h. The total duration of fever was 7.36 ± 4.48 h. Fever onset was earlier with penicillin G compared to ceftriaxone treatment (8.92 ± 4.98 h vs. 14.00 ± 5.90 h, 95% CI: 1.861 to 8.303, *P* < 0.05) and the time to defervescence was also shorter (16.35 ± 5.48 h vs. 20.80 ± 3.79 h, 95% CI: 1.085 to 7.848, *P* < 0.05). The time to reach peak temperature and the total duration of fever were comparable in the two arms (Table 2). These results suggest that penicillin G treatment was associated with an earlier onset of febrile reaction compared to ceftriaxone.

**Table 2:** Body temperature variation of 102 patients after penicillin G and ceftriaxone treatment

Variable		Overall (*N* = 102)	Antibiotic treatment		P value
			Penicillin G (*N* = 91)	Ceftriaxone (*N* = 11)	
Maximum temperature, mean (SD), ℃		38.81 (0.62)	38.81 (0.61)	38.75 (0.75)	0.755
Time to fever onset, mean (SD), hours	9.47 (5.29)		8.92 (4.98)	14.00 (5.90)	0.004
Time to peak temperature, mean (SD), hours		10.41 (4.80)	9.91 (4.45)	14.55 (5.79)	0.006
Time to defervescence, mean (SD), hours		16.83 (5.49)	16.35 (5.48)	20.80 (3.79)	0.002
Total duration of fever, mean (SD), hours		7.36 (4.18)	7.43 (4.13)	6.82 (4.73)	0.635

Data are shown for the 102 patients who developed fever during Jarisch–Herxheimer reaction; one JHR case did not develop fever and was excluded from temperature-based analyses. Time to fever onset: time from the first antibiotic dose to the first recorded temperature ≥ 37.2 °C. Time to peak temperature: time from the first antibiotic dose to the maximum temperature. Time to defervescence: time from the first antibiotic dose until temperature remained below 37.2 °C. Total duration of fever: time from fever onset to defervescence

The neuropsychiatric symptoms observed in penicillin G cohort included dysphoria (*n* = 10), disorganized speech (*n* = 8), abnormal behaviour (*n* = 6), consciousness disorder (*n* = 6), slow reaction (*n* = 4), hallucination (*n* = 4), seizures (*n* = 3), memory decline (*n* = 3), emesis (*n* = 2), urinary incontinence (*n* = 2), myoclonus (*n* = 1), encopresis (*n* = 1), aphasia (*n* = 1) (Fig. 3).

### Management strategies and outcome of JHR

JHR Management was symptoms-based. For temperatures below 38.5 °C in most cases, fever was managed with physical cooling measures such as using of ice packs, increasing hydration, and changing wet clothes. Indomethacin suppository was often utilized when temperature was above 38.5 °C. 76 (69.1%) patients received indomethacin suppositories, 1 (0.9%) patient received paracetamol, and 33 (30.0%) patients were managed with physical cooling. The febrile reaction was resolved in all patients, with a median duration of 7.36 h (Table 2).

Symptomatic treatment for neuropsychiatric symptoms were administered as follows: metoclopramide hydrochloride for emesis; diazepam, olanzapine and clonazepam for dysphoria; diazepam and dexamethasone sodium phosphate for consciousness disorder and seizure; clonazepam and olanzapine for slow reaction and memory decline; dexamethasone sodium phosphate and diazepam for consciousness disorder and seizure. Electrocardiogram (ECG) was performed in cases of consciousness disorder. Hallucination, abnormal behavior, disorganized speech, consciousness disorder, urinary incontinence, dysphoria, slow reaction, memory decline, emesis, aphasia, memory decline, and myoclonus could resolve spontaneously without specific management in some cases. While two cases were transferred to a general hospital due to persistent consciousness disorder, the neuropsychiatric symptoms in the other cases resolved within 2 to 7 days during antibiotic treatment (Table 3).

**Table 3:** Clinical characteristics and management strategies of the 23 patients manifested as neuropsychiatric symptoms

No.	Temp. (℃)	JHR neuropsychiatric symptoms	Management	Follow-up
1	37.9	Hallucination, Abnormal behavior, Disorganized speech, Emesis	Physical Cooling, Indomethacin suppository, Metoclopramide hydrochloride	Improved in day 4
2	38.7	Disorganized speech, Dysphoria	Physical Cooling, Indomethacin suppository, Diazepam	Improved in day 2
3	39.5	Hallucination, Disorganized speech	Physical Cooling, Indomethacin suppository	Improved in day 4
4	38.6	Abnormal behavior, Disorganized speech, Dysphoria	Physical Cooling, Indomethacin suppository, Diazepam, Olanzapine, Clonazepam	Improved in day 5
5	40.4	Consciousness Disorder, Urinary incontinence	Physical Cooling, Indomethacin suppository	Improved in day 2
6	39	Consciousness Disorder, Seizure	Physical Cooling, Diazepam, Dexamethasone Sodium Phosphate	Improved in day 2
7	39	Abnormal behavior, Disorganized speech, Dysphoria	Physical Cooling, Indomethacin suppository, Diazepam	Improved in day 2
8	39.8	Dysphoria	Physical Cooling, Indomethacin suppository	Improved in day 4
9	38.5	Slow reaction, Memory decline	Physical Cooling, Indomethacin suppository	Improved in day 2
10	39.2	Abnormal behavior, Dysphoria	Physical Cooling, Indomethacin suppository	Improved in day 3
11	38.5	Emesis, Aphasia	Physical Cooling, Indomethacin suppository	Improved in day 3
12	38.5	Slow reaction, Memory decline	Physical Cooling	Improved in day 3
13	39	Hallucination, Abnormal behavior, Disorganized speech	Physical Cooling, Indomethacin suppository	Improved in day 4
14	37.9	Dysphoria	Physical Cooling, Indomethacin suppository, Diazepam, Olanzapine, Clonazepam	Improved in day 3
15	40.3	Abnormal behavior, Myoclonus, Dysphoria	Physical Cooling, Indomethacin suppository	Improved in day 5
16	38.8	Consciousness Disorder, Encopresis, Urinary incontinence	Physical Cooling, Indomethacin suppository, ECG Monitoring	Transferred
17	37	Hallucination, Dysphoria	Monitoring	Improved in day 2
18	38.8	Slow reaction, Memory decline	Physical Cooling, Clonazepam, Olanzapine	Improved in day 7
19	39	Dysphoria, Slow reaction	Physical Cooling, Paracetamol	Improved in day 3
20	39.5	Disorganized speech, Dysphoria	Physical Cooling, Indomethacin suppository	Improved in day 2
21	38.2	Consciousness Disorder, Seizure	Dexamethasone Sodium Phosphate, Diazepam, ECG Monitoring	Improved in day 5
22	39.2	Disorganized speech, Consciousness Disorder	Physical Cooling, Indomethacin suppository	Improved in day 2
23	39	Consciousness Disorder, Seizure	Physical Cooling, Indomethacin suppository	Transferred

Temp.: The peak body temperature

Four general paresis cases with penicillin G treatment experienced severe JHR (Table 3): Case 6: A 51-year-old woman developed seizures and fever (38.5 °C) within 0.5 h post- penicillin G. Seizures lasted 3 min, consciousness returned 30 min with diazepam and dexamethasone, and symptoms resolved by day 2. Case 16: A 43-year-old man developed fever (38.8 °C) and mild consciousness 8 h post-penicillin G. Electrocardiogram monitor was performed, and he was transferred to a general hospital due to impaired consciousness, encopresis and urinary incontinence. Case 21: A 53-year-old man had three times seizures without fever, within 30 min of initiating penicillin G therapy. The symptoms resolved 5 days later with dexamethasone sodium phosphate and diazepam. Case 23: A 64-year-old man developed fever (39 °C) 9 h after treatment and triggered seizures twice 12 h later. He was transferred to a general hospital due to persistent consciousness disorder.

## Discussion

The JHR was first described by Jarisch in 1985 and later elaborated by Herxheimer in 1902 [23, 24]. JHR is a transient clinical phenomenon that occurs after the initiation of antibiotic treatment for spirochetal infections such as syphilis, leptospirosis, Lyme disease, and relapsing fever [25]. While its precise pathogenesis remains unclear, JHR is generally believed to result from the rapid destruction of spirochetes, leading to the release of toxins and cytokines, which drive systemic inflammation. JHR has been associated with early immune activation [26], elevated baseline C-reactive protein (CRP) levels [27], and increased CSF cytokines in neurosyphilis [28]. Patients with general paresis, CSF pleocytosis or a high CSF-VDRL titre were more likely to be at higher risk for JHR [13].

JHR without neuropsychiatric symptoms has been reported in 5.2%-54% neurosyphilis cases, while neuropsychiatric JHR occurs in 1.7%-12.7% of neurosyphilis subtypes [11–13]. In our study, the overall JHR incidence in neurosyphilis was 10.20% (103/1010). Comparatively, literatures reported an average JHR incidence rate of 28% in early syphilis, with rates of 56–79% in secondary syphilis, 37% in primary syphilis, and 7% in early latent syphilis [21, 29]. These findings indicate that JHR is less frequent in neurosyphilis than in early syphilis. Additionally, the median time to developing JHR in neurosyphilis in our study was 9.47 h, later than in the 5–6 h onset reported in early syphilis [21, 27, 30].

In the baseline characteristic analysis, CSF protein concentration was significantly higher in the penicillin G group. As CSF protein is a well-recognized marker of CNS inflammation, this finding may indicate a higher baseline inflammatory burden in these patients. Previous studies have reported that CSF protein concentration was not independently associated with the occurrence of JHR in neurosyphilis [13]. However, although higher CSF protein levels in the penicillin G group may suggest greater baseline inflammation, the influence of this difference on comparative JHR outcomes cannot be conclusively determined in this non-randomized study.

Penicillin remains the first-line treatment for all stages of syphilis, but it has been reported to trigger a higher frequency of JHR compared to doxycycline, azithromycin, erythromycin and tetracycline in early syphilis [19–21]. For instance, in one study of early syphilis, all JHR cases occurred in patients receiving penicillin (57/170), whereas no cases were observed in the doxycycline-treated group (0/12) [21]. Among HIV-positive patients with early syphilis, the incidence of JHR was significantly lower in those receiving azithromycin (14.1%) compared to those treated with benzathine penicillin (56.3%) [27]. A retrospective study further confirmed that JHR was significantly more common with penicillin (63%, 455/721) than with erythromycin or tetracycline (29%, 22/76) (*p* < 0.00005) [20]. Penicillin allergy represents an immune-mediated hypersensitivity reaction [31], whereas the Jarisch–Herxheimer reaction is believed to result from cytokine release following rapid spirochetal clearance [28]. Although certain characteristics associated with penicillin allergy (such as sex or age) have been reported [31, 32], current data are limited regarding whether penicillin allergy status itself influences the risk or clinical presentation of JHR.

Although penicillin G remains the first-line therapy for neurosyphilis, ceftriaxone is frequently used as an alternative—particularly in patients with penicillin G allergy or logistical constraints. Recent studies suggested comparable clinical and serological efficacy between intravenous ceftriaxone and high-dose penicillin G, albeit with limited sample sizes and heterogeneity across studies [22, 33]. In this study, we found that the overall incidence of JHR was similar between penicillin G and ceftriaxone arms, while penicillin G was associated with an earlier febrile onset, which has several practical implications. First, switching to ceftriaxone does not obviate the need for JHR surveillance. Second, the earlier onset with penicillin G may inform monitoring schedules and patient counseling—especially for patients with general paresis who are at higher risk for severe neuropsychiatric manifestations. Finally, when penicillin G is contraindicated or logistically challenging, ceftriaxone remains a reasonable alternative without apparent reduction in JHR risk; however, confirmation in prospective, preferably randomized, studies is required. These findings should be interpreted cautiously, as residual confounding related to treatment allocation and penicillin allergy status cannot be excluded.

JHR symptoms in early syphilis are generally mild, including fever, chills, lesion exacerbation, headache and myalgias, with severe cases being rare—only one report of life-threatening hypotension in secondary syphilis has been documented [21, 34–36]. While most JHR cases in neurosyphilis are harmless and self-limiting, severe cases can lead to seizures, status epilepticus (SE), generalized convulsive SE (GCSE), acute ischemic stroke or even fatal outcomes in general paresis and gummatous syphilis [15, 18, 28, 37–40]. In our study, 102 of 103 JHR patients presented with fever, while 23 (22.33%) exhibited neuropsychiatric symptoms—all of whom had general paresis. Four of these cases were complicated by seizures and prolonged loss of consciousness. These findings highlight the need for increased vigilance in managing general paresis cases, as they are at higher risk for severe JHR-related complications.

Currently, no pre-treatment has been definitively proven to prevent JHR. A clinical trial conducted on louse-borne relapsing fever found that pre-treatment with sheep anti-TNF-α Fab reduced the JHR incidence and severity by mitigating pro-inflammatory cytokines release [41], but this approach has not yet been validated in syphilis. Corticosteroids are sometimes used prophylactically, but their efficacy remains controversial. The 2020 European guidelines recommend corticosteroids for JHR prevention [9], whereas German guidelines do not [10]. None of the patients in our study received prophylactic corticosteroids or other pre-treatments. Given that most JHR cases are mild and corticosteroid effectiveness is limited [35, 41], routine prophylaxis may not be necessary [36, 42]. A randomized controlled study is warranted to determine whether corticosteroid can effectively prevent JHR.

Before initiating antibiotic treatment for neurosyphilis, patients should be informed about the JHR to reduce anxiety and encourage timely reporting of symptoms. Clinicians should differentiate JHR from allergic reactions, as JHR typically does not require treatment discontinuation [43]. Management is primarily supportive, focusing on symptom relief. Fever should be carefully controlled, particularly in patients with neurological conditions [43–45]. At present, antipyretics are recommended in guidelines for managing JHR-associated fever, however, there is no mention of specific antipyretic agents and regimen. It should be noted that the management of JHR-associated fever in our cohort sometimes included the empirical use of indomethacin suppositories. Indomethacin is a non-steroidal anti-inflammatory drug (NSAID) with potent antipyretic and anti-inflammatory effects via cyclooxygenase inhibition [44]. Paracetamol (acetaminophen) was also used in some cases for symptomatic relief. The empirical use of indomethacin suppositories for managing JHR-associated fever in neurosyphilis reflects local clinical practice rather than an evidence-based standard of care. Given the lack of robust evidence, our experience with indomethacin should be interpreted cautiously, and prospective studies are warranted to evaluate the optimal antipyretic regimen. Neuropsychiatric symptoms warrant close monitoring, with symptomatic management tailored to individual cases. For instance, olanzapine may be considered for dementia-related agitation [45], while carbamazepine is effective for seizure management [18].

This study has several limitations. Firstly, the allocation of the penicillin G and ceftriaxone cohort was non-randomized because the treatment regimen was determined according to STD guidelines. Penicillin was the first-line treatment, while ceftriaxone was treated for penicillin-allergic patients, which could introduce selection bias. Even though most baseline characteristics were broadly comparable between the two cohorts, residual baseline differences—including CSF protein concentration—cannot be fully excluded and may have influenced the observed outcomes, given the non-randomized study design. Secondly, the marked imbalance in sample size between the two groups may have reduced the statistical power and preclude robust multivariable adjustment. Thirdly, as a single-center retrospective study, the generalizability of our findings to other populations or healthcare settings is limited. Finally, our analyses were constrained by the accuracy and completeness of medical records, and some relevant confounders (for example, concomitant medications, comorbidities, or timing/dose of antipyretic interventions) could not be fully controlled. Therefore, multicenter, prospective randomized controlled studies are warranted to further validate our findings.

## Conclusion

JHR is a clinically relevant complication of neurosyphilis therapy, most commonly manifesting with fever and, in a subset of patients, neuropsychiatric symptoms. In our cohort, the overall incidence of JHR was comparable between penicillin G and ceftriaxone regimens, although penicillin G was associated with an earlier onset of febrile response. These findings underscore the need for vigilant monitoring for JHR irrespective of the antibiotic chosen. Given the retrospective, single-center, and non-randomized design of this study, our results should be interpreted with caution; larger multicenter prospective studies are needed to confirm these observations and to inform optimal monitoring and management strategies.

## Data Availability

The datasets generated during and/or analyzed during the current study are available from the corresponding author on reasonable request.

## Abbreviations

CNS: Central nervous system
JHR: Jarisch-Herxheimer reaction
WHO: World Health Organization
ECDC: European Centre for Disease Prevention and Control
EU/EEA: European Union/European Economic Area
IV: Intravenous
STD: Sexually transmitted disease
CSF: Cerebrospinal fluid
VDRL: Venereal Disease Research Laboratory
TPPA: Treponema pallidum particle agglutination
WBC: White blood cell
t: temperature
TRUST: Toluidine red unheated serum test
WBC: White blood cell
SD: Standard deviation
IQR: Interquartile range
ECG: Electrocardiogram
SE: Status epilepticus
GCSE: Generalized convulsive SE
TNF-α: Tumour necrosis factor-α
